# Ghrelin Inhibits Intestinal Epithelial Cell Apoptosis Through the Unfolded Protein Response Pathway in Ulcerative Colitis

**DOI:** 10.3389/fphar.2021.661853

**Published:** 2021-03-10

**Authors:** Lin Zhang, Jian Cheng, Jie Shen, Sheng Wang, Chuanyong Guo, Xiaoming Fan

**Affiliations:** ^1^ Department of Gastroenterology, Jinshan Hospital, Fudan University, Shanghai, China; ^2^ Center of Emergency and Intensive Care Unit, Jinshan Hospital, Fudan University, Shanghai, China; ^3^ Department of Gastroenterology, Jinshan Hospital, Fudan University and Institutes of Biomedical Sciences, Fudan University, Shanghai, China; ^4^ Department of Gastroenterology, Tenth People’s Hospital, Tongji University, Shanghai, China

**Keywords:** ghrelin, ulcerative colitis, apoptosis, unfolded protein response pathway, intestinal epithelial cell

## Abstract

Ulcerative colitis (UC) is a type of inflammatory bowel disease (IBD) that occurs in the lining of the rectum and colon. Apoptosis of the intestinal epithelial cells (IECs) is common in active UC patients. Ghrelin is reported to be downregulated in apoptosis of IECs induced by tumor necrosis factor-α (TNF-α). Therefore, we hypothesized that ghrelin might play an antiapoptotic role in UC progression, which was investigated using *in vitro* and *in vivo* studies. The TNF-α-treated Caco-2 cell model and mouse colitis model induced by dextran sulfate sodium (DSS) or 2,4,6-trinitrobenzenesulfonic acid (TNBS) were established and employed. We found that ghrelin could inhibit the apoptosis of Caco-2 cells induced by TNF-α, which could be disturbed by [D-lys3]-GHRP-6, the antagonist of ghrelin receptor GHS-R1a. Similarly, in the DSS- and TNBS-induced mouse colitis models, ghrelin could also protect intestinal tissues from apoptosis in DSS- and TNBS-induced colitis depending on GHS-R1a. Furthermore, ghrelin modulated the unfolded protein response (UPR) pathway and regulated the expressions of caspase-3, BAX, and Bcl-2, which contributed to the inhibition of cell apoptosis. In conclusion, ghrelin protects IECs from apoptosis during the pathogenesis of colitis by regulating the UPR pathway.

## Introduction

Ulcerative colitis (UC) is a type of inflammatory bowel disease (IBD) that occurs in the lining of the rectum and colon (Wang et al., 2020). The main pathological features of UC are weight loss, rectal bleeding, mucosal ulceration, and epithelial barrier disruption (Rapa et al., 2021). Due to the unclear pathogenesis of UC, there are no effective drugs adopted in the clinical treatment of this kind of disease. Thus, exploring the mechanism and new therapeutic drugs for UC is urgent.

The intestinal epithelial barrier (IEB) separates the intestinal microorganism from the intestinal tissue, and its dysfunction is reported to be associated with UC (Maloy and Powrie, 2011). To keep the homeostasis of IEB, apoptosis and proliferation of the intestinal epithelial cells (IECs) should achieve a dynamic balance. It is reported that excessive apoptosis induced by endoplasmic reticulum (ER) stress is common in the development of UC (Luo and Cao, 2015; Zeng et al., 2015). ER stress activates the unfolded protein response (UPR) pathway and promotes the chaperone glucose-regulated protein 78 (GRP78, also known as BIP and HSPA5) to dissociate from the ER transducer sensors’ luminal domain to stabilize protein folding (Chung et al., 2011; Cao and Kaufman, 2012). Meanwhile, this ER stress signaling system can induce transcription of C/EBP homologous protein (CHOP), a transcription factor that binds with other transcription factors and induces proapoptotic genes (Puthalakath et al., 2007). When the capacity of the UPR is overwhelmed by its proteostasis, apoptosis will be triggered (Ron and Walter, 2007), involving the apoptosis-related proteins, Bcl-2, BAX, and BAK (Hetz et al., 2020).

Currently, studies on the pathogenesis of UC are mostly focused on immunity and apoptosis. As an important cytokine, the role of TNF-α in the inflammatory process of UC has been demonstrated. Increasing evidence has proved the genetic association between TNF-α and UC (Smillie et al., 2019). Moreover, increased TNF-α levels have been noted in patients with UC (Ren et al., 2018). It has been reported that TNF-α administration could promote interstitial cells of Cajal (ICC) apoptosis and UC progression, along with the downregulation of ghrelin (Ren et al., 2018), indicating that ghrelin could play a protective role against UC by inhibiting cell apoptosis.

Ghrelin was originally identified in gastric cells to regulate nutrient sensing and appetite (Pereira et al., 2017). It works via binding to its receptor, growth hormone secretagogue receptor (GHS-R), which is proved to be broadly expressed by immune cells. Ghrelin could function in immune systems to suppress inflammation (Hattori, 2009). However, it is still unclear whether ghrelin could improve inflammation damage during UC.

The TNF-α-treated Caco-2 cell model and mouse colitis model induced by dextran sulfate sodium (DSS) or 2,4,6-trinitrobenzenesulfonic acid (TNBS) are well-established models for UC investigation. With these models, we found that ghrelin inhibited the apoptosis of Caco-2 cells induced by TNF-α, which could be disturbed by [D-lys3]-GHRP-6, the antagonist of GHS-R1a (Patel et al., 2012). Also, ghrelin protected intestinal tissues from IECs apoptosis in DSS- and TNBS-induced colitis, depending on GHS-R1a. Furthermore, ghrelin modulated the UPR pathway and regulated the expression of caspase-3, BAX, and Bcl-2, which may contribute to the protective role of ghrelin in UC progression.

## Materials and Methods

### Cell Culture

Human colon epithelial adenocarcinoma Caco-2 cells were provided by Cell Bank of Chinese Academy of Science (Shanghai, China). The cells were maintained at 37°C in Dulbecco's Modified Eagle's Medium (DMEM) with 10% fetal bovine serum (Gibco, Grand Island, NY, USA).

### Cell Treatment

Caco-2 cells were seeded at 1.0 × 10^5^ cells/well in 6-well plates and incubated overnight to adhesion. Ghrelin (ProSpec, Israel) was diluted and then added to the plates at final concentrations of 0.01, 0.1, 1, and 10 μmol/L for 1 h before treatment with 100 ng/ml TNF-α (Sigma, St. Louis, MO, United States). For GSH-R1a blocking array, [D-lys3]-GHRP-6 (antagonist of GSH-R1a, Tocris, United Kingdom) (Patel et al., 2012) was used to pretreat the cells for 1 h before the ghrelin administration. Cells were stained with Hoechst 33258 for apoptosis analysis.

### Colitis Mouse Model

Animal experiment protocols were approved by the Institute Animal Use and Care Committee of Jinshan Hospital. Male C57BL/6J mice (eight weeks old) were provided by SLAC Laboratory Animal Co. (Shanghai, China). A batch of mice was treated with 2.5% w/v DSS (ICN Biomedical, Australia) provided ad libitum for nine days. Ghrelin (25–250 μg/kg) was intraperitoneally administered for ten days. The mice were randomly divided into six groups as follows (n = 10): control group (received tap water only), model group (colitis induced by DSS), treatment (25 μg/kg) group (colitis induced by DSS + 25 μg/kg ghrelin), treatment (125 μg/kg) group (colitis induced by DSS + 125 μg/kg ghrelin), treatment (250 μg/kg) group (colitis induced by DSS + 250 μg/kg ghrelin), and treatment + antagonist group (colitis induced by DSS + 250 μg/kg ghrelin + 9.4 mg/kg [D-lys3]-GHRP-6).

Another batch of mice was anesthetized with isoflurane and then injected with 2.5% w/v TNBS (ICN Biomedical, Australia) in 50% ethanol into the colon via a cannula. Ghrelin (25-250 μg/kg) was intraperitoneally administered for ten days. The mice were randomly divided into six groups as follows (n = 10): control group (received 50% ethanol only), model group (colitis induced by TNBS), treatment (25 μg/kg) group (colitis induced by TNBS + 25 μg/kg ghrelin), treatment (125 μg/kg) group (colitis induced by TNBS + 125 μg/kg ghrelin), treatment (250 μg/kg) group (colitis induced by TNBS + 250 μg/kg ghrelin), and treatment + antagonist group (colitis induced by DSS + 250 μg/kg ghrelin + 9.4 mg/kg [D-lys3]-GHRP-6).

### Cell Apoptosis Staining

Mice were sacrificed on day 10 using overdosed sodium pentobarbital, and then distal colon tissues were collected and fixed with 4% paraformaldehyde overnight. Intestinal tissues were embedded and cut into 5 μm sections and then stained using the TUNEL kit (Beyotime Biotechnology Institute, Nantong, China).

### RNA Isolation and Quantitative Real-Time RT-PCR (Rt-qPCR)

Total RNA was extracted from Caco-2 cells and intestinal tissues with TRIzol reagent (Ambion, United States). qPCR measurement was performed using One-Step SYBR® PrimeScript™ RT-PCR kit (Takara, Dalian, China). The sequences of the primers used in RT-qPCR were as follows: GRP78 5′-TCT​CAG​ATC​TTC​TCC​ACG​GC-3′ and 5′-CTT​CAG​CTG​TCA​CTC​GGA​GA-3’; CHOP 5′-TCA​CTA​CTC​TTG​ACC​CTG​CG-3′ and 5′-ACT​GAC​CAC​TCT​GTT​TCC​GT-3’; BAX 5′-GTG​GTG​GAG​GAA​CTC​TTC​AGG​G-3′ and 5′-GCC​GGT​TCA​GGT​ACT​CAG​TCA​T-3’; Bcl-2 5′-TTT​TGC​TAC​AGG​GTT​TCA​TCC​A-3′ and 5′-GTG​TCC​ACG​TCA​GCA​ATC​ATC-3’; GAPDH 5′-ATG​GGT​GTG​AAC​CAC​GAG​A-3′ and 5′-CAG​GGA​TGA​TGT​TCT​GGG​CA-3’. Relative gene expression levels were determined using the 2^–ΔΔCT^ method.

### Western Blotting Analysis

Caco-2 cells or frozen intestinal tissues were lysed with RIPA buffer (Beyotime Biotechnology Institute, China). All the antibodies used in this study, including GRP78, CHOP, total and phosphor-JNK, ERK, eIF2, pro- and cleaved caspase-12 and caspase-3, BAX, Bcl-2, and *β*-actin were purchased from Cell Signaling Technology (Cambridge, MA, United States).

### Statistical Analysis

All the experiments were performed at least three times. Data were shown as means ± standard deviation (SD) and analyzed by SPSS 18 software (SPSS, Chicago, IL, United States). Differences among the groups were analyzed by Student’s *t*-test or one-way analysis of variance followed by Tukey’s *post hoc* test. *p* < 0.05 indicated significant differences.

## Results

### Ghrelin Inhibits the Apoptosis of Caco-2 Cells Induced by TNF-α Through GHS-R1a

**FIGURE 1:**
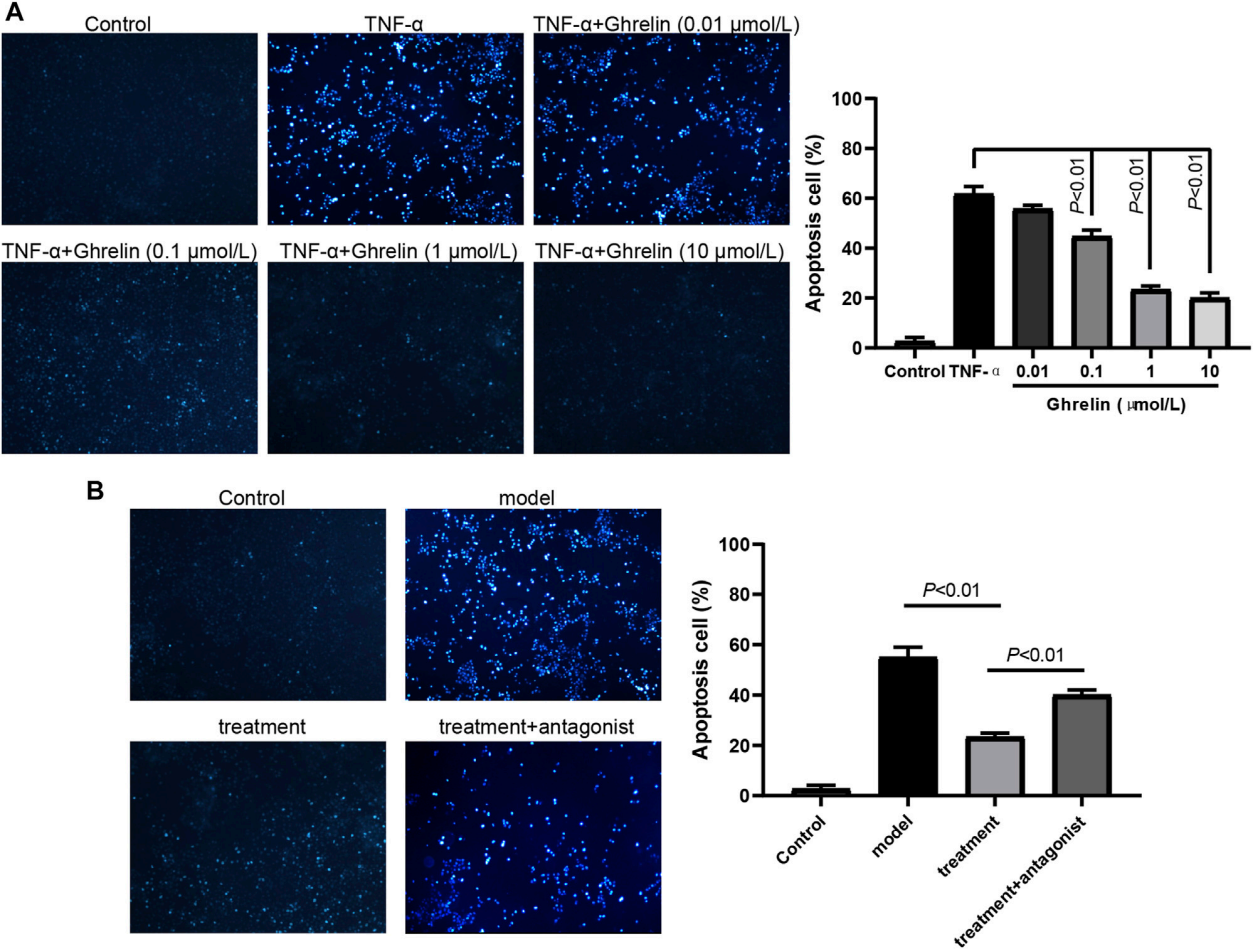
Ghrelin inhibits TNF-α-induced apoptosis of Caco-2 cells through GHS-R1a. **(A)** Caco-2 cells were treated with ghrelin, and apoptosis was observed by Hoechst 33258 staining. Ghrelin inhibited apoptosis at the dose from 0.01 μmol/L to 10 μmol/L. Data were shown as means ± SD (n = 3). **(B)** Caco-2 cells were treated with ghrelin in addition to [D-lys3]-GHRP-6, and apoptosis was observed by Hoechst 33258 staining. Data were shown as means ± SD (n = 3).

As previously reported, TNF-α could induce Caco-2 cells’ apoptosis by downregulating the expression of ghrelin (Ren et al., 2018). Therefore, we hypothesized that ghrelin administration could inhibit the apoptosis of Caco-2 cells induced by TNF-α. To prove our hypothesis, TNF-α and ghrelin were employed to cotreat the Caco-2 cells with ghrelin final concentration at 0.01–10 μmol/L, respectively. The cell apoptosis staining assay showed that the percentage of TNF-α-induced Caco-2 cell apoptosis was more than 60% and that ghrelin inhibited TNF-α-induced Caco-2 cell apoptosis in a dose-dependent manner (Figure 1A). To explore whether this effect of ghrelin is mediated by GHS-R1a, we employed GHS-R1a antagonist, [D-lys3]-GHRP-6, in our study. We found that [D-lys3]-GHRP-6 dramatically abrogated the inhibition of cell apoptosis caused by ghrelin (Figure 1B). These results show that ghrelin plays an antiapoptotic role in TNF-α-treated Caco-2 cells via GHS-R1a.

### Ghrelin Inhibits the Apoptosis of Caco-2 Cells Induced by TNF-α Through UPR Pathway *In Vitro*

**FIGURE 2:**
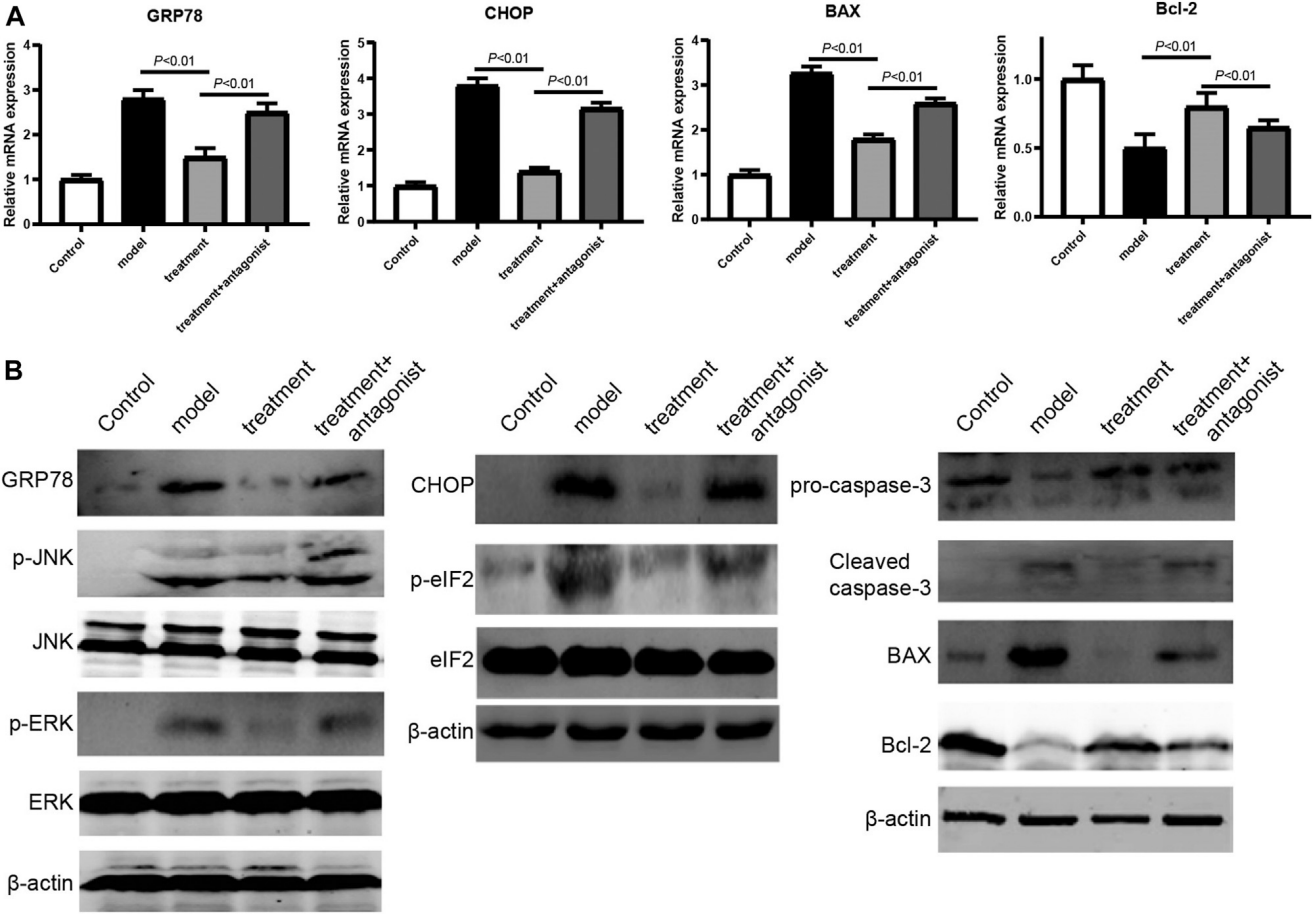
Ghrelin regulated UPR pathway and apoptosis in Caco-2 cells. **(A)** RT-qPCR analysis of the expression levels of GRP78, CHOP, BAX, and Bcl-2. Data were shown as means ± SD (n = 3). **(B)** Western blot analysis of GRP78, phospho-JNK, and phosphor-ERK. **(C)** Western blot analysis of CHOP and phospho-eIF2. **(D)** Western blot analysis of caspase-3, BAX, and Bcl-2. Representative blots from three independent experiments were shown.

To explore the possible mechanisms underlying the antiapoptotic effect of ghrelin, we focused on the UPR pathway, which regulates the balance between cell survival and apoptosis. The RT-qPCR assay showed that the expression of UPR pathway members (GRP78 and CHOP) and proapoptotic gene BAX was significantly increased in TNF-α-treated Caco-2 cells, whereas the antiapoptotic gene Bcl-2 was downregulated in TNF-α-treated Caco-2 cells (Figure 2A). When ghrelin was used to treat the TNF-α-treated Caco-2 cells, the expression trends of GRP78, CHOP, BAX, and Bcl-2 were reversed, suggesting that ghrelin inhibited the apoptosis of Caco-2 cells induced by TNF-α by regulating the UPR pathway and apoptosis-related genes (Figure 2A). We also found that the regulation of GRP78, CHOP, BAX, and Bcl-2 by ghrelin could be disturbed by its receptor antagonist [D-lys3]-GHRP-6 (Figure 2A). Furthermore, western blotting was performed to verify this finding. When the cells were treated with ghrelin, the expression of GRP78 and CHOP, the phosphorylation of ERK, JNK, and eIF2, and the levels of proapoptosis proteins caspase-3 and BAX were all decreased and the expression of Bcl-2 was increased (Figure 2B). These results indicate that ghrelin inhibits the apoptosis of Caco-2 cells induced by TNF-α through the UPR pathway *in vitro*.

### Ghrelin Protects Colitis Tissues Cells From Apoptosis *In Vivo*

**FIGURE 3:**
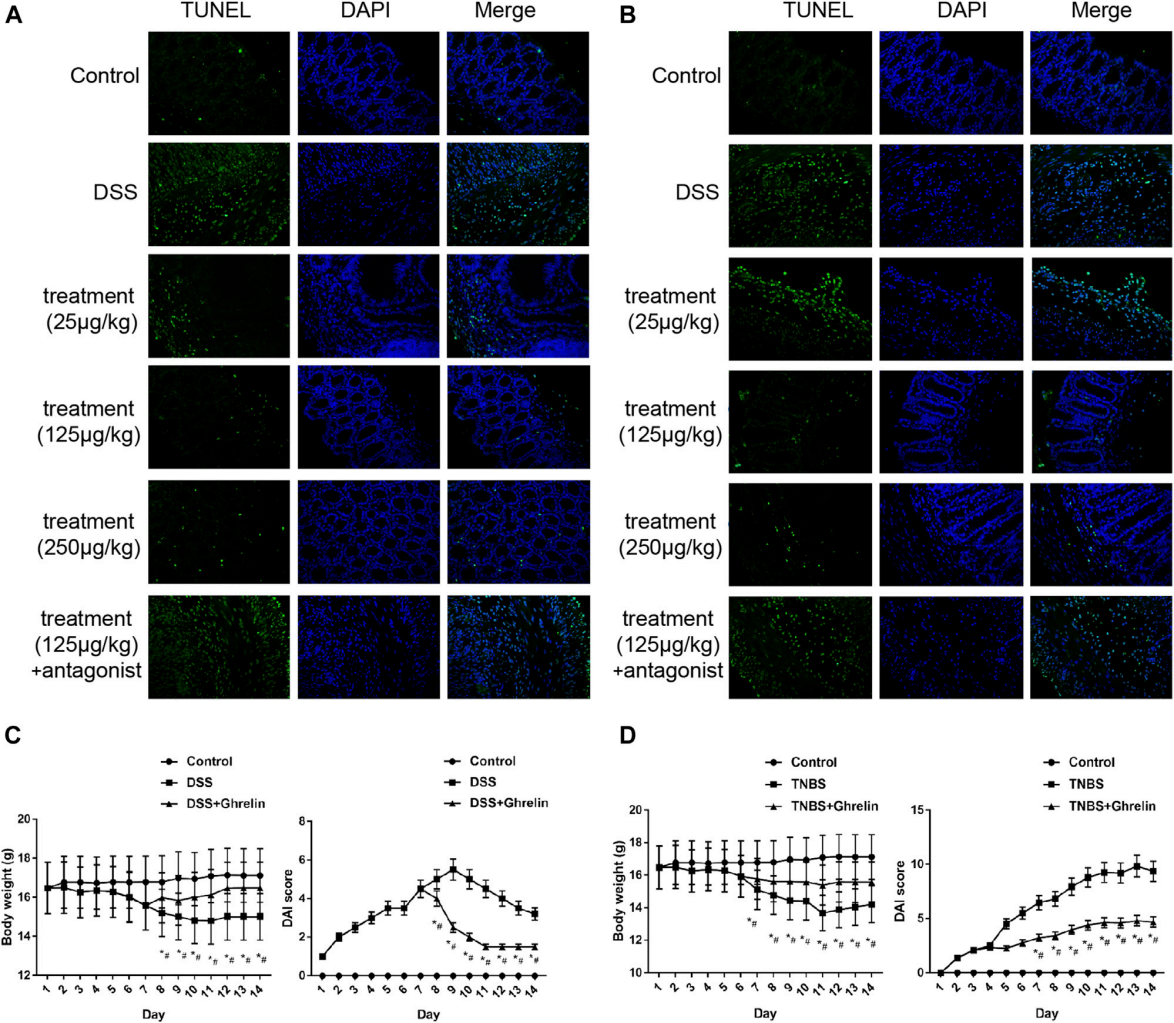
Ghrelin reduced apoptosis in DSS- and TNBS-induced colitis in mice. **(A)** and **(B)** Antiapoptotic effects of ghrelin could be observed at 125 and 250 μg/kg in the DSS model and at 25, 125, and 250 μg/kg in the TNBS model, and GHS-R1a antagonist [D-lys3]-GHRP-6 abrogated antiapoptotic effects of ghrelin. **C**. Body weight and the DAI score in the DSS model group. **D**. Body weight and the DAI score in the TNBS model group. Representative blots from ten mice in each group were shown. *p < 0.05 DSS (or TNBS) vs. control, #*p* < 0.05 DSS (or TNBS)  + Ghrelin vs. DSS (or TNBS).

To verify the antiapoptotic role of ghrelin *in vivo*, we used ghrelin to treat the DSS- and TNBS-induced colitis models, respectively. As previously reported, the colon of mice treated with DSS or TNBS displayed more significant apoptosis (Arab et al., 2021; Salem et al., 2021) (Figures 3A, 3B). Compared with the treatment group, ghrelin reduced the colon cell apoptosis in a dose-dependent manner, and such an antiapoptotic effect of ghrelin could be reversed by [D-lys3]-GHRP-6 (Figures 3A,B). Furthermore, we measured and calculated the body weight and the disease activity index (DAI) (Cooper et al., 1993) of mice in the control group, model group, and treatment group. As a result, the model group showed lower mice weight and higher DAI scores than those of the control group. Meanwhile, the treatment group showed higher mice weight and lower DAI scores than those in the model group (Figures 3C,D). These results further confirm that ghrelin could protect colitis tissue cells from apoptosis in DSS- and TNBS-treated mice.

### Ghrelin Inhibited Apoptotic Through Modulating UPR Pathway *In Vivo*

**FIGURE 4:**
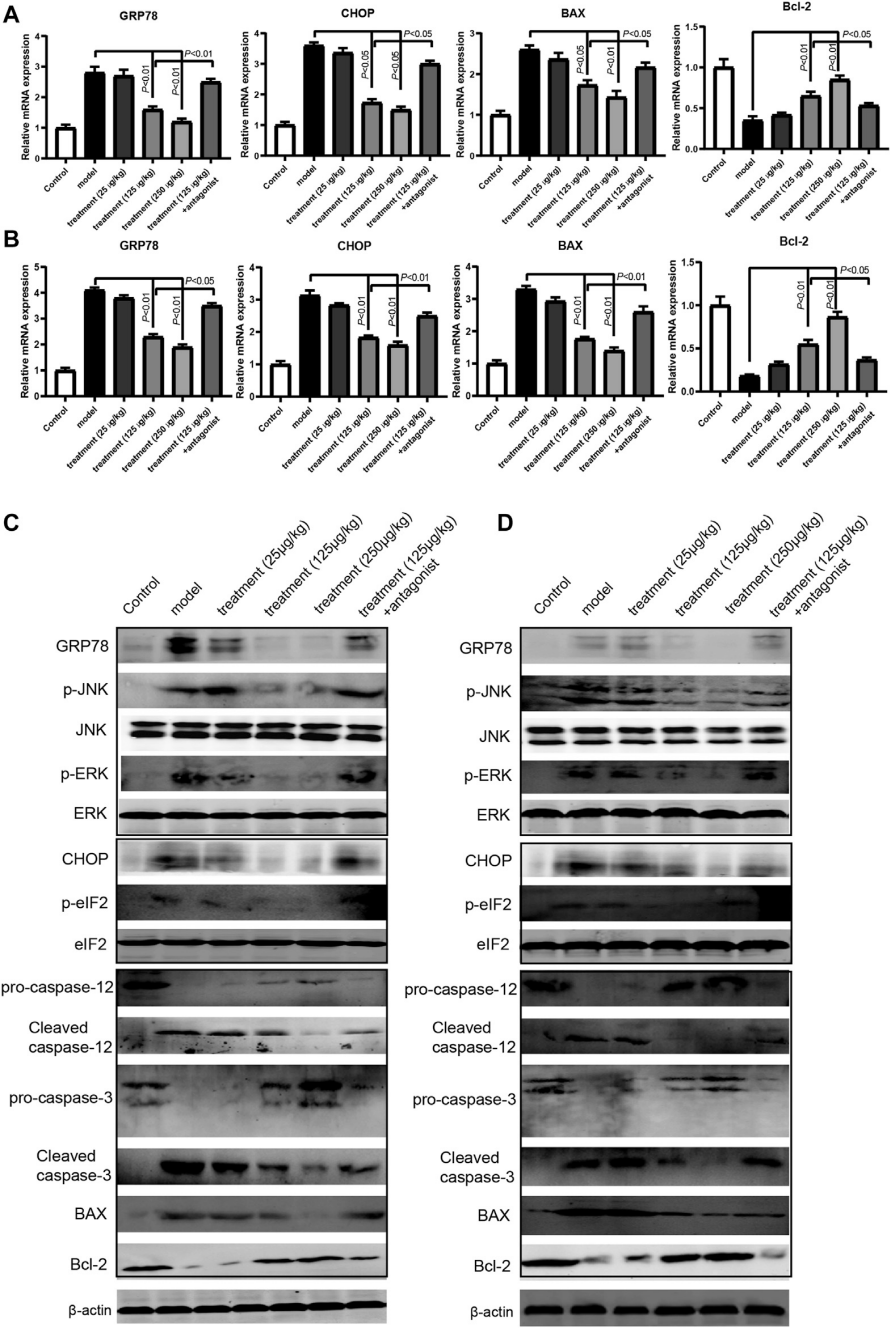
Ghrelin regulated UPR pathway and apoptosis in intestine tissues of model mice. **(A**) and **(B)** RT-qPCR analysis of the expression levels of GRP78, CHOP, BAX, and Bcl-2 in the DDS model and TNBS model. Data were shown as means ± SD (n = 3). **p < 0.01 compared to DSS or TNBS group. &*p* < 0.05 compared to ghrelin (250 μg/kg) group. &&*p* < 0.01 compared to ghrelin (250 μg/kg) group. **(C)** Western blot analysis of GRP78, phospho-JNK, phosphor-ERK, CHOP, phospho-eIF2, caspase-12, caspase-3, BAX, and Bcl-2 in the DSS model. **(D)** Western blot analysis of GRP78, phospho-JNK, phosphor-ERK, CHOP, phospho-eIF2, caspase-12, caspase-3, BAX, and Bcl-2 in the TNBS model.

To explore our findings *in vivo*, we measure the mRNA and protein expression of the members of the UPR pathway and relevant apoptosis molecules in intestine tissues of the mice colitis model. The RT-qPCR results showed that ghrelin reduced the mRNA expression of GPR78, CHOP, and BAX and increased the mRNA expression of Bcl-2 in a dose-dependent manner in mice colitis models, and such an effect of ghrelin could be reversed by [D-lys3]-GHRP-6 (Figures 4A,B). Similarly, the western blotting results showed that ghrelin reduced the expression of GRP78 and CHOP, decreased ERK, JNK, and eIF2 phosphorylation, decreased the levels of proapoptosis proteins caspase-3 and BAX, and increased antiapoptosis protein Bcl-2 level in intestinal tissues (Figures 4C,D). These results indicate that ghrelin plays a protective role against colitis by modulating the UPR pathway.

## Discussion

UC is a common intestinal bowel disease characterized by intestinal epithelial injury, including extensive epithelial cell death, mucosal erosion, ulceration, and crypt abscess formation (Lu et al., 2020; Wang et al., 2020). Several signaling pathways, including the NF-κB/NLRP3 inflammasome pathway (Zeng et al., 2020), RIPK pathways (Garcia-Carbonell et al., 2019), and the JAK/STAT pathway (Zundler and Neurath, 2016), contribute to disease progression. In this study, we found that ghrelin could protect intestinal barrier in the colitis model induced by DSS via the UPR pathway. We employed the Caco-2 cell apoptosis model *in vitro* and mouse colitis models *in vivo* to confirm the protective role of ghrelin against colitis. It was illustrated that ghrelin inhibited apoptosis of Caco-2 cells induced by TNF-α, which could be disturbed by [D-lys3]-GHRP-6. Moreover, the antiapoptotic effect of ghrelin may be attributed to its regulation of the protein expressions in the UPR pathway, such as caspase-3, BAX, and Bcl-2. In mouse colitis models, we found that ghrelin exhibited protective effects on colitis in a GHS-R1a-dependent manner, and the effects may be mediated by the UPR components, such as caspase-3, BAX, and Bcl-2, in intestine tissues.

Notably, ghrelin exhibited protective effects on Caco-2 cell apoptosis in a dose-dependent manner, consistent with the protective effects of ghrelin reported in a previous study (Ercan et al., 2015). However, ghrelin had no significant effects on Caco-2 cell apoptosis at both dosage of 0.01 μmol/L and a higher dose of 10 μmol/L. Furthermore, in the *in vivo* conditions, the effective concentration of ghrelin was 25 μg/kg in the TNBS model and 125 μg/kg in the DSS model, consistent with the previous studies on the application of ghrelin in colitis models induced by TNBS and DSS (Gonzalez-Rey et al., 2006; Konturek et al., 2009).

GHS-R1a and GHS-R1b are two subtypes of ghrelin receptors (Smith et al., 2001). GHS-R1a mediates the effects of ghrelin by secreting growth hormone, but the mechanism of GHR-R1b remains unclear (Muccioli et al., 2004; Gauna et al., 2005; Negroni et al., 2014). In our study, it was demonstrated that [D-lys3]-GHRP-6 abrogated the antiapoptotic effects of ghrelin on colitis both *in vitro* and *in vivo*, suggesting that GHS-R1a is a mediator in the beneficial effects of ghrelin on colitis. However, the effects of ghrelin on colitis were not completely reversed by [D-lys3]-GHRP-6, indicating that GHR-R1b may also contribute to beneficial effects of ghrelin on colitis.

Preclinical and clinical studies indicate that the UPR pathway is implicated in the pathogenesis of colitis (Kaser et al., 2010). The abnormal UPR pathway may induce epithelial cell death, activate proinflammatory response, and damage the mucosal barrier, contributing to the development of colitis (Cao, 2015). Our *in vivo* and *in vitro* studies showed that ghrelin could downregulate the expression of the UPR pathway molecules GRP78 and CHOP and proapoptosis proteins caspase-3 and BAX, upregulate the antiapoptosis protein Bcl-2, and decrease the phosphorylation of ERK, JNK, and eIF2. Moreover, recent studies showed that the UPR pathway was related to autophagy (Giraud-Billoud et al., 2018; Hooper et al., 2019). We did not evaluate other possible mechanisms such as autophagy that is reported to contribute to the protective effect of ghrelin since previous studies have shown that NFκB and MAPK pathways may mediate the effects of ghrelin (Ma et al., 2001; Li et al., 2004). Further investigations into the contribution of different pathways for the protective effects of ghrelin will provide new data for developing novel therapeutic targets.

## Conclusion

We demonstrated that ghrelin protected IECs from apoptosis during the pathogenesis of colitis, perhaps by inhibiting the UPR pathway.

## Data Availability

The original contributions presented in the study are included in the article/Supplementary Material; further inquiries can be directed to the corresponding authors.
